# Maternal Ferritin Levels during Pregnancy and ADHD Symptoms in 4-Year-Old Children: Results from the INMA–INfancia y Medio Ambiente (Environment and Childhood) Prospective Birth Cohort Study

**DOI:** 10.3390/ijerph17217704

**Published:** 2020-10-22

**Authors:** Loreto Santa-Marina, Nerea Lertxundi, Ainara Andiarena, Amaia Irizar, Jordi Sunyer, Amaia Molinuevo, Sabrina Llop, Jordi Julvez, Andrea Beneito, Jesús Ibarluzea, Liher Imaz, Maite Ferrin

**Affiliations:** 1 Spanish Consortium for Research on Epidemiology and Public Health (CIBERESP), Instituto de Salud Carlos III, C/Monforte de Lemos 3–5, 28029 Madrid, Spain; ambien4ss-san@euskadi.eus (L.S.-M.); jordi.sunyer@isglobal.org (J.S.); au-molinuevo@euskadi.eus (A.M.); llop_sab@gva.es (S.L.); jordi.julvez@isglobal.org (J.J.); mambien3-san@euskadi.eus (J.I.); 2 Biodonostia, Epidemiology and Public Health Area, Environmental Epidemiology and Child Development Group, 20014 San Sebastian, Spain; nerea.lertxundi@ehu.eus (N.L.); ainara.andiarena@ehu.eus (A.A.); 3 Public Health Division of Gipuzkoa, Basque Government, 20013 San Sebastian, Spain; l-imazgoienetxea@euskadi.eus; 4 Faculty of Psychology, University of the Basque Country (UPV/EHU), Avenida Tolosa 70, 20018 San Sebastian, Spain; 5 Faculty of Medicine and Nursing, University of the Basque Country (UPV/EHU), Barrio Sarriena s/n, 48940 Leioa, Spain; 6 Hospital del Mar Research Institute, 08003 Barcelona, Spain; 7 ISGlobal—Instituto de Salud Global de Barcelona–Campus MAR, PRBB, 08003 Barcelona, Spain; 8 Epidemiology and Environmental Health Joint Research Unit, FISABIO–Universitat Jaume I–Universitat de València, 08003 València, Spain; beneito_and@gva.es; 9 Institut d′Investigació Sanitària Pere Virgili (IISPV), Hospital Universitari Sant Joan de Reus, 43204 Reus, Spain; 10 Biodonostia, Epidemiology and Public Health Area, Epidemiology of Chronic and Communicable Diseases Group, 20014 San Sebastian, Spain; 11 Haringey Child and Adolescent Mental Health Service, Barnet, Enfield and Haringey NHS Mental Health Trust, London N15 3TH, UK; maiteferrin@yahoo.es; 12 Recognition Health, London W1G 9RU, UK

**Keywords:** ferritin status, pregnancy, ADHD symptoms, childhood

## Abstract

Ferritin status during prenatal brain development may influence the risk of attention deficit and hyperactivity disorder (ADHD) symptoms in childhood. We investigated the association of maternal ferritin in pregnancy and ADHD-like symptoms in offspring. A total of 1095 mother-child pairs from three birth cohorts of the INMA Project (Spain) were studied. Maternal plasma ferritin in pregnancy was measured at 11.57 weeks of gestation. Children′s ADHD-like symptoms at ages 4–5 years were assessed using the ADHD Rating Scale-IV. The count model of the zero-inflated Poisson regression model showed a significant inverse association between ferritin (continuous variable) and inattention, β = −0.19 (−0.32, −0.07), for boys. Comparing ferritin level by tertiles, significant differences were observed between the first tertile ([1.98, 20.92]) and the second ([20.92, 38.79]) and third tertiles ([38.79, 216.5]) (mg/L).The number of symptoms was lower for those in the third tertile, β = −0.3 (−0.55, −0.5), and for those in the second one, β = −0.37 (−0.6, −0.14). The model stratification by sex also showed this inverse association for boys only, β = −0.21 (−0.34, −0.08). No associations were found between ferritin level and hyperactivity or total ADHD symptoms. High ferritin levels during pregnancy show a protective association with child inattentive-type ADHD symptoms.

## 1. Introduction

Attention deficit/hyperactivity disorder (ADHD) is the most frequent childhood-onset neuropsychiatric condition, with an estimated worldwide prevalence of approximately 5% in school-aged children [1]. ADHD symptoms tend to persist into adulthood in as many as 65% of cases [2]. 

Despite ADHD being the most studied neuropsychiatric condition in child psychiatry worldwide [3], the etiological factors [4] are not well understood [5]. No specific etiology has been identified for ADHD, and findings are consistent with a multifactorial model [6]. Indeed, the disorder is likely to be due to a complex combination of environmental, genetic and biological factors. The range of etiologies related to prenatal and perinatal risk factors, genetics and neurobiological deficits that have been proposed may all be involved in the pathophysiology of ADHD [7]. 

The human brain is highly sensitive to environmental exposure occurring during particular periods of vulnerability [8,9]. In early life, biological brain development is highly active and any factors enhancing or disturbing this process could have permanent effects on brain function [10]. It is also established that both genetic and a wide range of environmental factors, including physical, biological, psychological and social factors, are able to modulate brain structure and its function by interacting with genes and expression mechanisms (i.e., epigenetic determinants) [11]. 

In recent years, it has been suggested that iron deficiency (ID) may contribute to behavioral and cognitive dysfunctions [12]. Iron is an essential trace metal, which plays a central role in many essential brain functions [13]. Animal models show that early iron deficiency may lead to structural and functional brain abnormalities including alterations in dopamine metabolism, energy metabolism and myelination [14]. Several case-control studies have reported a significant association between iron deficiency and low scores in tests assessing mental, social and motor development in infants, as well as a lower intelligence quotient [15], poor learning performance and impaired neuropsychological functions (e.g., poor spatial memory) [13].

Further evidence supports the hypothesis that a lack of brain iron might contribute to the pathophysiology of ADHD [16,17]. Firstly, iron is a cofactor of enzymes necessary for the synthesis and catabolism of the monoaminergic neurotransmitters including dopamine [18], which has been linked to ADHD [7,19]. Secondly, ID is associated with a decrease in dopamine transporter expression [20], and the corresponding gene is linked to a genetic vulnerability for ADHD [21]. Thirdly, ID may lead to dysfunction in the basal ganglia [22], which again are believed to play a significant role in the pathophysiology and expression of ADHD [5]. 

The role of serum ferritin levels as a reliable measure of iron stores in body tissues, including the brain, in children with ADHD has been studied in recent years. A meta-analysis [23] and a systematic review [24] have found contradictory findings for the relationship between ferritin levels and ADHD, and therefore the extent to which serum ferritin correlates with brain iron levels remains unclear [25,26]. In addition, maternal ferritin levels and their effects on child neuropsychological development have been less studied. A prospective population-based study in a rural province in Vietnam, which enrolled 497 pregnant women at 12–20 weeks of gestation and followed them up with their infants until six months postpartum, found that lower ferritin levels were associated with a poorer cognitive function and worse social and emotional development in the children [27]. Similarly, a recent study based on health and population register data from the Stockholm Youth Cohort evaluated 532,232 women with ID diagnosed during the first 30 weeks of pregnancy and found that anemia diagnosed up to this point in pregnancy was associated with an increased risk of autism spectrum disorder (ASD), ADHD and intellectual disability in offspring [12].

Therefore, the objective of the present study was to evaluate the relationship between prenatal ferritin levels with infant ADHD symptoms. For that, the association between ID during pregnancy measured by ferritin levels in maternal serum and ADHD symptoms in children aged 4–5 years was assessed. Findings might have important implications for clinical practice, regarding prenatal iron supplementation.

## 2. Materials and Methods 

### 2.1. Subjects

**Figure 1:**
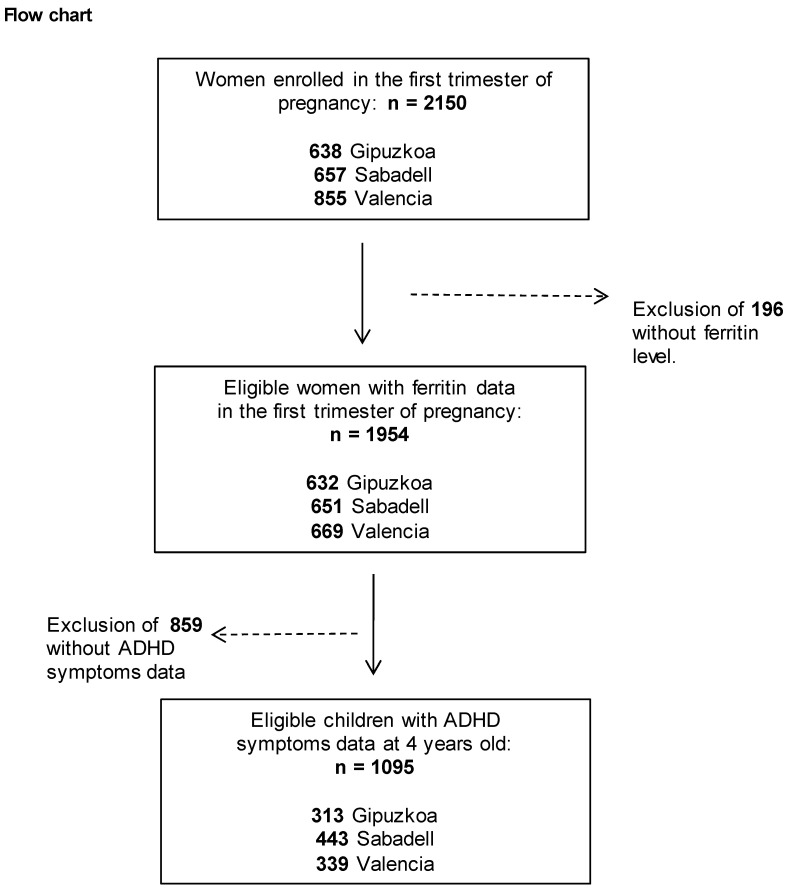
Maternal ferritin levels (mg/L) were assessed during the first trimester of pregnancy. The study was approved by the ethics committees of the centers involved in the study, and written informed consent was obtained from the parents of all children.

The INMA (INfancia y Medio Ambiente, Childhood and Environment) Project [28], a multicenter birth cohort study, was established between 2003 and 2008 in 3 regions of Spain, namely: Gipuzkoa (Basque Country), Sabadell (Catalonia) and Valencia. Participant recruitment and follow-up procedures have been reported in detail elsewhere (Guxens et al., 2012). A total of 2644 eligible women were recruited during prenatal visits in the first trimester of pregnancy. Inclusion criteria were: ≥16 years of age, singleton pregnancy and intention to deliver at the reference hospital; and exclusion criteria were: women having any communication problems that might hinder their participation in the study, fetuses having malformations and pregnancies having been achieved by assisted conception. Women were followed up during pregnancy, and their children were enrolled at birth and followed up until 4–5 years of age. The population finally studied was composed of 1095 pregnant women from the general population resident in Sabadell (N = 443), Valencia (N = 339) or Gipuzkoa (N = 313) and their children at 4 years of age (Figure 1).

### 2.2. Ferritin Measurement

Maternal whole blood samples were collected during pregnancy (mean [SD] 13.3 [1.5] weeks of gestation) by venipuncture under fasting conditions and stored between −70 and −80 °C until analysis. Maternal plasma ferritin concentrations were quantified in samples from Gipuzkoa and Sabadell cohorts by time-resolved fluorescence immunoassay (DELFIA Ferritin kit A069–101), at the Gipuzkoa Public Health Laboratory, and in samples from the Valencia cohort by immunoturbidimetry (Beckman Coulter AU analysers) at La Fe hospital.

### 2.3. Child and Family Characteristics

Data on maternal and child characteristics were collected through two questionnaires administered during face-to-face interviews at different points in the first and third trimester of pregnancy, at birth, at 14 months after birth and when children reached 4–5 years of age. We used data on maternal country of origin (Spain/other), maternal social class (manual or lower class [IV and V] and non-manual/skilled or higher class [I–III]), maternal education (primary or lower, secondary and university), maternal age, maternal mental health (mental problems, yes/no), maternal intelligence quotient, parity, smoking during pregnancy (yes/no), alcohol intake during pregnancy (yes/no), pre-pregnancy body mass index (BMI) (underweight = BMI <18.5; normal weight = BMI 18.5–25; overweight = BMI 25–30; and obesity = BMI >30 kg/m^2^) and breastfeeding (weeks of any breastfeeding). Anthropometric measures at birth and the sex of the infant were obtained from the child’s medical records. Deliveries before 37 weeks of gestation were defined as preterm births. Gestational age was calculated from the date of the last menstrual period reported at recruitment and confirmed using ultrasound examination at week 12 of gestation. Whole blood samples were collected by venipuncture of cord vessels before the placenta was delivered for the measurement of mercury in the newborn. At 14 months of age, various types of data related to the children were collected including daycare attendance, birth order among siblings (first/not first), whether they were living with their parents (both/only one) and the number of people in the household. The exact age of the child was also recorded at the time of the assessment of ADHD-like symptoms.

### 2.4. ADHD Symptoms

Child ADHD symptoms at 4 years of age were assessed in the period 2008–2013 using the ADHD Rating Scale-IV developed by DuPaul, Power and Anastopoulos [29] completed by the child’s classroom teacher, which reflects the Diagnostic and Statistical Manual of Mental Disorders 4th edition Criteria for ADHD (ADHD-DSM-IV; [30]). The scale comprises 18 ADHD symptoms and is designed to evaluate inattention (9 symptoms) and hyperactivity/impulsivity (9 symptoms). Each symptom is rated using a 4-point scale: 0 = “not at all”, 1 = “just a little”, 2 = “pretty much” and 3 = “very much”.

Scores were summed to provide continuous measures of inattention, hyperactivity/impulsivity and total ADHD symptoms. The presence of symptoms in the clinical range was estimated by coding ratings of 0 and 1 as “symptom absent” and ratings of 2 and 3 as “symptom present”. The good psychometric characteristics of this measurement in the INMA project have been reported previously [31].

### 2.5. Statistical Analysis

First, we compared socio-demographic characteristics of mothers and children across the three cohorts studied (Gipuzkoa, Sabadell and Valencia). The difference in the mean number of symptoms was analyzed using the Student’s t-test (for two samples) and one-way analysis of variance (more than two samples). 

Due to the excess of zeros in symptom ratings, the association between ferritin level and symptoms was analyzed using zero-inflated Poisson regression models. This type of model has two parts, a Poisson count model and a logit model for predicting excess zeros. The level of ferritin was entered in the models in two ways: as a continuous variable and categorized in tertiles (Tertile 1: [1.98, 20.92]; Tertile 2: (20.92, 38.79]; and Tertile 3: (38.79, 216.5] (mg/L)) to study the trend across the tertiles. Initially, variables with a *p*-value ≤ 0.20 in the bivariate analysis were added to the base models (ferritin level and ADHD symptoms). These variables were maintained in the models if they changed the magnitude of the main effects by more than 10% or were significant. The final model was adjusted for: cohort (Gipuzkoa, Sabadell or Valencia), maternal smoking during pregnancy (yes/no), alcohol intake during pregnancy (g/day), pre-pregnancy BMI and social class (manual/non-manual), the child’s sex, age at the time of the test and whether they were living with parents (both/only one) and the week of sample collection for ferritin analysis. For the count model of the association of inattention symptoms with ferritin levels, the interaction between ferritin and sex was significant, and hence results are shown separately for boys and girls. The final model was also stratified by cohort and sex of the child. Statistical analyses were conducted using R version 3.6.1. 

## 3. Results

Overall, 1095 eligible mother-child pairs were included in this study (Figure 1). The socio-demographic characteristics of mothers and children across the three cohorts (Gipuzkoa, Sabadell and Valencia) are summarized in Table 1. The Valencian cohort had higher levels of ferritin and an earlier week of blood sample collection than the Gipuzkoa and Sabadell cohorts. Mothers recruited in Gipuzkoa were the oldest, breastfed for the longest and had the highest level of education and social class, lowest BMI and lowest percentage of foreign mothers. Regarding lifestyle during pregnancy, Valencia had the highest rates of smoking and alcohol consumption among the mothers. In addition, children from Valencia had the highest levels of Hg in umbilical cord blood. The mean ages of mothers and children at assessment of ADHD-like symptoms were 30.9 years and 4.9 years, respectively. Compared with women excluded, those included in the analysis were older, had higher levels of education and social class, breastfed for longer and tended to smoke less during pregnancy (Supplementary Table S1).

The mean maternal ferritin level was 35.9 mg/L (standard deviation: 26.81 mg/L). In the univariate analyses, the total ADHD symptom score was higher in boys than girls. Further, total ADHD symptom scores were higher in children of mothers with higher BMI, lower alcohol intake, worse mental health and lower education and social class, who smoked and did not breastfeed (Table 2). 

Considering ferritin levels as a continuous variable, the count model of the zero-inflated Poisson regression model showed a significant inverse association between ferritin levels and inattention symptoms, β = −0.19 (−0.32, −0.07), in boys. In addition, comparing ferritin levels by tertiles for boys, there were significant differences between the first and second tertile, β = −0.3 (−0.55, −0.05), and the first and third tertile, β = −0.37 (−0.6, −0.14), with a significant trend (*p* = 0.002). In contrast, no significant association was found between ferritin level and hyperactivity/impulsivity or total ADHD symptom scores (Table 3).

The stratification of the count model by the sex of the child also showed an inverse association between ferritin level and inattention symptoms for boys, both when taking ferritin levels continuously, β = −0.21 (−0.34, −0.08), and also when comparing the individuals by tertiles: the expected change in the number of symptoms was lower for those in the second tertile, β = −0.32 (−0.57, −0.06), and for those in the third one, β = −0.4 (−0.63, −0.16) (p for trend 0.001). In contrast, the count model for inattention showed a positive association for the girls in the third tertile of ferritin levels, β = 0.58 (0.02, 1.13), compared with those in the first one (p for trend 0.012). Similarly, when considering the total number of ADHD symptoms, a negative association was observed for the boys in the third tertile, β = −0.22 (−0.39, −0.06). The zero-inflation model for ADHD symptoms showed a positive association, β = 0.6 (0.05, 1.16), for girls in the second tertile and a positive association was also observed when taking ferritin level as a continuous variable, β = 0.39 (0.08, 0.69) (Table 4).

## 4. Discussion

The present study assessed the association between maternal ferritin levels during pregnancy and ADHD symptoms in their offspring when the children reached 4–5 years of age. Significant inverse associations were observed between maternal ferritin level during pregnancy and symptoms of inattention in boys. The stratification by sex of the count model also showed this negative association between inattention symptoms in boys and ferritin levels, both analyzed continuously and by tertiles. In contrast, in girls, a positive association was observed with ferritin levels in the third tertile compared with the first tertile. This positive association was also found for the zero-inflated model of total ADHD symptoms in girls.

There is a large body of evidence about iron deficiency during pregnancy and maternal and fetal outcomes [32]. Two recent systematic reviews and meta-analyses suggest that ADHD is related to lower serum ferritin levels in children [33,34]. Nonetheless, few studies have been published so far analyzing the association between maternal ferritin levels during pregnancy and long-term effects on child neurodevelopment. Our results support the evidence from another prospective population-based study, which showed a negative association between iron deficiency during pregnancy and cognitive, social and emotional development [27], and a longitudinal study [35], where low umbilical cord serum ferritin levels were associated with poorer performance in mental and psychomotor tests in children at 5 years of age. Along similar lines, the Stockholm Youth Cohort that evaluated 532,232 children found an association between prenatal iron deficiency and risk of neurodevelopmental disorders, including ASD, ADHD and intellectual disability later in life [12]. Anemia diagnosed within the first 30 weeks of pregnancy was related to an increased risk of ASD, ADHD and especially intellectual disability.

### 4.1. The Role of Iron During Neurodevelopment

The involvement of iron in several biological processes has been described previously [36]. Nevertheless, studies on the effects of iron on brain function are limited and they are based on either animal or cell culture studies or inferred from magnetic resonance imaging data in humans. These studies can be divided into those that focus on: (i) oligodendrocyte metabolism [37,38,39], (ii) monoamine metabolism [20,39,40,41] and (iii) GABA metabolism [42]. 

Serum ferritin reflects the status of iron stores in the body: bone marrow stores are only depleted when the level falls below 10 ng/mL and then anemia develops. The role of iron deficiency with and without anemia and developmental/cognitive deficits has been demonstrated in various neurologic and developmental disorders in both laboratory and clinical studies [43]. As iron is preferentially used for the synthesis of blood hemoglobin when the body is short of iron, the brain may become rapidly iron-depleted when intake is reduced, even if the individual is not showing clinical symptoms of anemia [44]. 

Human studies have shown that iron deficiency and iron-deficiency anemia in infants are associated with behavioral impairment [45,46], but the periods of brain development most susceptible to iron deficiency have not been established. Iron depletion of the brain occurs in rats within several weeks of being on a low-iron diet and is replenished with refeeding very quickly when the iron depletion occurs in the neonatal and postneonatal periods; this contrasts to intrauterine iron deficiency in which the effects of iron depletion appear irreversible [47]. Similarly, in humans, the consequences appear reversible with treatment when iron deficiency occurs in preschool and school-aged children. For example, it is known that iron supplementation may improve attention and concentration irrespective of baseline iron status [48]. 

ADHD is a complex multifactorial condition in which multiple genes and environmental factors act together to affect individual risk and contribute to an ADHD phenotype and clinical presentation. The heritability of ADHD was estimated at 72% based on 1316 child and adolescent twin studies [49]. On the other hand, the effects of the environment and gene-environment interactions are hard to separate from purely genetic contributions. 

Various models have indicated that dopamine is a key element of ADHD pathophysiology. First, dopamine is associated with the modulation of psychomotor activity and executive functions, which are core features in ADHD. Second, molecular genetic studies of ADHD have focused on the genes involved in dopaminergic function, especially the dopamine D4 receptor gene and the dopamine transporter gene (DAT1) [50]. Third, the dopamine transporter is the main target for medications that are widely used by individuals with ADHD. Finally, iron is also a cofactor of tyrosine hydroxylase, the rate-limiting enzyme for dopamine synthesis. Therefore, brain iron stores might influence dopamine metabolism and subsequently affect various behavioral features, in particular, those described in people with ADHD symptoms. 

### 4.2. ADHD in Relation to Maternal Characteristics

In our study, total ADHD and inattention symptom scores were also predicted by maternal years of study, social class, maternal smoking during pregnancy, maternal BMI and breastfeeding. ADHD has traditionally been associated with a range of indicators of social and economic disadvantage including poverty, housing tenure, maternal education, income, lone parenthood and younger motherhood; the association of ADHD with socioeconomic disadvantage has found several potential explanatory pathways often operating through differential exposure [51]. Such exposures could be perinatal, prenatal or occur during childhood. We observed a direct relationship between maternal smoking during pregnancy and ADHD symptoms in the child. Children whose mother smoked during pregnancy showed more inattention, hyperactivity and total ADHD symptoms. A systematic review and meta-analysis reported a positive association between smoking during pregnancy and a higher risk of ADHD [52]. Similarly, several studies have also observed this increasing risk [53,54], although other research suggests that this association may be due to failure to control for confounding familial factors. In fact, the association was lost in some studies performing sibling-matched analysis [55,56]. Our results suggest that BMI is correlated with ADHD symptoms in early childhood, with more ADHD symptomatology being observed in children whose mothers were obese. In a longitudinal study, and after adjusting for potential causal pathway factors including pregnancy weight gain, gestational diabetes, breastfeeding duration, postpartum depression and child′s birth weight, children whose mothers were severely obese before pregnancy had a higher risk of adverse developmental outcomes including ADHD [57]. In another longitudinal birth cohort, pre-pregnancy obesity was also associated with problem behaviors in offspring including internalizing behaviors, externalizing behaviors and attention problems [58].

Finally, in our study, breastfeeding was not a predictor of total ADHD symptoms or inattention in 4 to 5-year-old children according to the multivariate model, though it was significantly correlated with ADHD in the bivariate analysis. A robust body of research literature has reported on the short-term medical, developmental and emotional benefits of breastfeeding for infants and toddlers [59,60]. A recent systematic review has also pointed out the long-term neurodevelopmental outcomes, suggesting that children who breastfeed for longer than six months have better cognitive outcomes and a lower risk of developing attention deficit/hyperactivity disorder [60]. 

### 4.3. Sex Differences

In accordance with previous studies, we observed sex differences in our results regarding inattention, hyperactivity/impulsivity and ADHD symptoms. Differences have been reported previously [61,62,63], with the diagnosis of ADHD being more common in male children and adolescents, with a male-to-female ratio ranging from 2:1 to 10:1 [64]. In line with this, in the present study, boys had more symptoms than girls. On the other hand, some authors have found a higher rate of the inattentive subtype among girls [65]. 

Overall, some of these differences may be partly explained by diagnostic and ascertainment biases. Nonetheless, it is likely that most are due to sex biological differences [66]. There is evidence of gender differences in brain activity in patients with ADHD [67]. Interestingly, in our study, ferritin levels seem to be impacting males, while females remain unaffected. This could suggest different etiologies or risk factors underlying similar clinical presentations. 

A hypothesis that could explain the sex-specific association of ferritin and ADHD symptoms is related to acetylcholinesterase (AChE) activity. AChE activity has been associated with deficits in neurodevelopment, particularly attention, inhibition and memory, in boys but not in girls [68]. In laboratory studies performed in rats, higher iron levels were associated with lower AChE activity in the brain compared to controls. These results suggested that, at least in part, iron-induced cognitive deficits were related to a dysfunction of cholinergic neural transmission in the brain [69]. 

Another explanation could be related to mitochondrial function in brain cells, since mitochondrial proteins have been related to neurodevelopmental abnormalities and to the pathophysiology of ADHD [70]. Iron overload in brain cells causes oxidative damage, mitochondrial dysfunction and cell death [71,72]. It has been shown that the effects of oxidative stress in neuron mitochondria during hypoxia-ischemia injury are sex-specific, causing sex-dependent survival rates of neurons [73]. Several recent studies have detected sex-dependent differences in mitochondrial proteins related to ADHD symptoms. Lee et al. (2019) observed different expression patterns of mitochondrial HtrA2 serine protease in boys and girls with ADHD [74], and similarly, Hwang et al. (2019) found that different mitochondrial DNA haplogroups had gender-dependent different functions in ADHD patients [75]. Interestingly, these studies suggested a greater sensitivity to mitochondrial protein changes in girls, in accordance with a previous study that demonstrated a higher susceptibility of female neuron mitochondria during hypoxia-ischemic injury [73]. In fact, Lee et al. (2019) suggested a potential female-specific mitochondria pathway in ADHD [74]. In our study, higher ferritin levels were positively associated with ADHD in girls, which could be due to a higher sensitivity of girls to increased iron (ferritin) levels in the brain and its negative effects on mitochondrial activity. Indeed, mitochondria biogenesis is more noteworthy in developing female brains [76].

Finally, in our study, ferritin levels in maternal serum at week 12 of pregnancy predicted symptoms of inattention; interestingly, maternal ferritin level was not a predictor of hyperactive and impulsivity symptoms. Previous studies have already suggested how different ADHD presentations or subtypes may correlate with different etiopathological models [77]. Our results also support the view that iron status may play a role in the cognitive deficits associated with ADHD, in particular, inattention and executive function. As cognitive impairment and executive function in ADHD are strong predictors of poorer outcome in children with ADHD [78], future studies could explore whether low maternal ferritin levels are also correlated with poorer-executive function offspring with ADHD in the follow-up. In addition, future studies should consider the differences found between males and females and the role of iron deficiency in the two groups separately. 

This study has a number of limitations which should be recognized. First, the study explored ADHD symptoms assessed using a rating scale based on DSM-IV criteria; however, a confirmed clinical diagnosis was not available. In addition, we only have a single measurement of ferritin during pregnancy, and it might not be a representative measure of ferritin levels throughout pregnancy. Moreover, ferritin levels in children were not measured, and therefore the study relies solely on maternal ferritin levels in the first trimester of pregnancy. 

On the other hand, this study has considerable strengths such as the large sample size covering different geographical regions, which increases the external validity, the prospective design and the inclusion of several confounding variables in the statistical models.

In summary, this study makes a new contribution to understanding the association between ferritin levels in mothers during pregnancy and ADHD symptoms in their children. There is a need for more research to clarify the mechanisms of action of iron and neurotransmitters in relation to cognitive function. 

## 5. Conclusions

Maternal ferritin levels during pregnancy are independently associated with ADHD symptoms in 4 to 5-year-old offspring. More specifically, maternal ferritin levels seem to influence inattention symptoms and specifically in boys, whereas girls seem to be more protected. Other maternal variables including BMI and education level are also associated with ADHD and inattention symptoms.

## Figures and Tables

**Table 1:** Descriptive characteristics of the study sample.

		GIPUZKOA		SABADELL		VALENCIA		*p*-Value *	TOTAL	
		N = 313		N = 443		N = 339			N = 1095	
Variables		N (%)	Mean (SD)	N (%)	Mean (SD)	N (%)	Mean (SD)		N (%)	Mean (SD)
*Child characteristics*										
Sex	Female	157 (50.16)		217 (48.98)		166 (48.97)		0.939	540 (49.32)	
	Male	156 (49.84)		226 (51.02)		173 (51.03)			555 (50.68)	
	*Missing*	*0 (0)*		*0 (0)*		*0 (0)*			*0 (0)*	
Preterm	No	301 (96.17)		425 (95.94)		320 (94.4)		0.168	1046 (95.53)	
	Yes	11 (3.51)		12 (2.71)		18 (5.31)			41 (3.74)	
	*Missing*	*1 (0.32)*		*6 (1.35)*		*1 (0.29)*			*8 (0.73)*	
Sibling order	Not first	143 (45.69)		188 (42.44)		147 (43.36)		0.669	478 (43.65)	
	First	170 (54.31)		255 (57.56)		192 (56.64)			617 (56.35)	
	*Missing*	*0 (0)*		*0 (0)*		*0 (0)*			*0 (0)*	
Lives with mother/father	Both	295 (94.25)		426 (96.16)		332 (97.94)		0.488	1053 (96.16)	
	Only one	3 (0.96)		5 (1.13)		7 (2.06)			15 (1.37)	
	*Missing*	*15 (4.79)*		12 (2.71)		*0 (0)*			*27 (2.47)*	
Number of people who they live with	*Missing*	*15 (4.79)*	2.58 (0.72)	12 (2.71)	2.57 (0.79)	*0 (0)*	2.58 (0.76)	0.997	*27 (2.47)*	2.57 (0.76)
Child’s age at the time of the test	*Missing*	*119 (1.74)*	4.41 (0.22)	*1 (0.09)*	4.44 (0.26)	*0 (0)*	5.92 (0.32)	**<0.001**	*20 (1.83)*	4.90 (0.75)
Breastfeeding	*Missing*	*13 (4.15)*	29.33 (20.14)	*1 (0.23)*	27.48 (19.64)	*0 (0)*	23.04 (19.3)	**<0.001**	*14 (1.28)*	26.6 (19.82)
Hg (μg/l) in umbilical cord	*Missing*	*35 (11.18)*	*9.6 (5.83)*	*117 (26.41)*	8.35 (6.53)	*95 (28.02)*	15.34 (11.6)	**<0.001**	*247 (22.56)*	10.77 (8.65)
*Maternal characteristics*										
Ferritin level (log scale)	*Missing*	*0 (0)*	3.28 (0.74)	*0 (0)*	3.25 (0.79)	*0 (0)*	3.45 (0.68)	**<0.001**	*0 (0)*	3.32 (0.75)
Maternal blood collection week	*Missing*	*0 (0)*	13.33 (1.31)	*2 (0.45)*	13 (1.8)	*0 (0)*	8.09 (4.23)	**<0.001**	*2 (0.18)*	11.57 (3.58)
Alcohol intake during pregnancy	*Missing*	*0 (0)*	0.19 (0.47)	*0 (0)*	0.37 (1.07)	*0 (0)*	0.41 (1.19)	**0.009**	*0 (0)*	0.33 (0.98)
Pre-pregnancy BMI	*Missing*	*0 (0)*	22.9 (3.35)	*0 (0)*	23.87 (4.71)	*0 (0)*	23.8 (4.43)	**0.004**	*0 (0)*	23.57 (4.29)
Age	*Missing*	*0 (0)*	31.58 (3.48)	*1 (0.23)*	30.66 (4.1)	*0 (0)*	30.64 (4.06)	**0.002**	*1 (0.09)*	30.92 (3.94)
Mental health (14 months after birth)	*Missing*	*39 (12.46)*	9.23 (3.33)	*27 (6.09)*	9.9 (3.95)	*339 (100)*	−	**0.021**	*405 (36.99)*	9.64 (3.72)
Parity	*Missing*	*0 (0)*	0.53 (0.64)	*2 (0.45)*	0.49 (0.65)	*0 (0)*	0.5 (0.64)	0.705	*2 (0.18)*	0.51 (0.64)
Smoking during pregnancy	No	269 (85.94)		379 (85.55)		264 (77.88)		**<0.001**	912 (83.29)	
	Yes	34 (10.86)		58 (13.09)		75 (22.12)			167 (15.25)	
	*Missing*	10 (3.19)		6 (1.35)		*0 (0)*			16 (1.46)	
Country of birth	Spain	305 (97.44)		402 (90.74)		318 (93.81)		**0.001**	1025 (93.61)	
	Other	8 (2.56)		39 (8.8)		21 (6.19)			68 (6.21)	
	*Missing*	*0 (0)*		2 (0.45)		*0 (0)*			2 (0.18)	
Social class	No manual	190 (60.7)		247 (55.76)		157 (46.31)		**<0.001**	594 (54.25)	
	Manual	123 (39.3)		196 (44.24)		182 (53.69)			501 (45.75)	
	*Missing*	*0 (0)*		*0 (0)*		*0 (0)*			*0 (0)*	
Education level	Primary	34 (10.86)		104 (3.48)		90 (26.55)		**<0.001**	228 (20.82)	
	Secondary	116 (37.06)		190 (42.89)		155 (45.72)			461 (42.1)	
	University	162 (51.76)		146 (32.96)		94 (27.73)			402 (36.71)	
	*Missing*	*1 (0.32)*		*3 (0.68)*		*0 (0)*			4 (0.37)	

* One-way ANOVA for mean differences and chi-squared test for categorical variables.

**Table 2:** Percentiles 75–90–95, mean and standard deviation for inattention, hyperactivity and ADHD symptoms.

		Inattention						Hyperactivity						ADHD					
		Percentiles			Mean	sd	*p* *	Percentiles			Mean	sd	*p* *	Percentiles			Mean	sd	*p* *
Variables																			
*Child characteristics*																			
Sex	Female	0	1	2	0.33	1.13	**<0.001**	0	1	3	0.43	1.23	**<0.001**	0	2.1	5	0.76	1.97	**<0.001**
	Male	1	4	6	0.89	1.98		1	3	5	0.76	1.76		2	6	9	1.65	3.3	
Preterm	No	0	2	5	0.61	1.63	0.768	0	2	4	0.6	1.54	0.362	1	4	8	1.21	2.76	0.848
	Yes	1	2	3	0.68	1.54		1	2	2	0.46	0.87		2	3	5	1.15	1.89	
Sibling order	Not first	0	2	4.15	0.63	1.61	0.884	0	2	4	0.59	1.48	0.871	1	4	7	1.21	2.66	0.997
	First	0	2	5	0.61	1.67		0	2	4	0.6	1.57		1	5	8	1.21	2.84	
Lives with mother/father	Both	0	2	5	0.61	1.63	0.760	0	2	4	0.59	1.53	**0.020**	1	4	7.4	1.2	2.75	0.342
	Only one	0	0	2.1	0.47	1.81		0	0.6	1.3	0.2	0.56		0	1.2	3.8	0.67	2.09	
Number of people who they live with	≤2	0	2	4	0.56	1.55	0.318	0	2	3.15	0.57	1.49	0.693	1	4	7.15	1.13	2.63	0.415
	> 2	0	2	5	0.67	1.73		0	2	4	0.61	1.55		1	4	7.55	1.27	2.87	
Child´s age at the time of the test	≤4.50	0	1	3	0.46	1.4	**0.001**	0	2	3	0.49	1.7	**0.018**	1	3	5	0.95	2.39	**0.001**
	> 4.50	0	3	5.65	0.79	1.87		0	3	4.65	0.72	1.37		1	5	9	1.51	3.11	
Breastfeeding (weeks)	No	1	4.7	6	1.02	2.17	**0.010**	0	3	5	0.78	1.83	**0.028**	2	8	10	1.8	3.54	**0.007**
	0–16	0	2	5	0.67	1.67		1	3	5	0.79	1.91		1	5	8.35	1.47	3.13	
	16–24	0	2.5	5	0.66	1.79		0	2	4	0.57	1.4		1	5	6.5	1.24	2.92	
	>24	0	2	3.7	0.49	1.39		0	2	3	0.48	1.28		1	4	6	0.97	2.24	
Hg (μg/l) in umbilical cord	≤8,2	0	2	5	0.64	1.690	0.608	0	2	3	0.58	1.58	0.791	1	4	7.75	1.22	2.87	0.653
	>8.2	0	2	4	0.59	1.590		0	2	4	0.55	1.46		1	4	7	1.14	2.68	
*Maternal characteristics*																			
Cohort	Gipuzkoa	0	1	3	0.42	1.29	**0.012**	0	1	2	0.35	1.18	**0.004**	0	3	5	0.77	2.22	**0.002**
	Sabadell	0	2	5	0.62	1.62		1	2	4	0.69	1.6		1	4.8	7.9	1.31	2.8	
	Valencia	0	3	6	0.8	1.92		0	3	4.1	0.7	1.69		1	5	9	1.5	3.1	
Ferritin level (log scale)	≤3.37	0	2	5	0.61	1.67	0.955	0	2	4	0.62	1.49	0.636	1	4	8	1.23	2.75	0.819
	>3.37	0	2	4.8	0.62	1.61		0	2	4	0.57	1.57		1	4.6	7	1.19	2.77	
Maternal blood collection week	≤12	0	2	5	0.67	1.72	0.300	0	2	4	0.61	1.53	0.824	1	5	8	1.27	2.82	0.462
	>12	0	2	4	0.56	1.55		0	2	4	0.59	1.53		1	4	7	1.15	2.7	
Alcohol intake during pregnancy	≤0.0201	0	3	5.8	0.71	1.850	**0.019**	0	2	4	0.67	1.65	**0.052**	1	5	9	1.39	3.12	**0.013**
	>0.0201	0	2	4	0.49	1.300		0	2	3	0.5	1.34		1	4	6	0.99	2.18	
Pre-pregnancy BMI	Underweight	0	2	2.9	0.51	1.1	**<0.001**	0	1	2.9	0.49	1.39	0.073	0.5	4	4.9	1	2.01	**<0.001**
	Normal weight	0	2	4	0.49	1.44		0	2	4	0.54	1.42		1	4	6	1.03	2.46	
	Overweight	0	3	6	0.78	1.92		1	2	3.4	0.69	1.68		1	5	9.4	1.47	3.31	
	Obese	2	6	7.2	1.42	2.44		1	4	5.6	0.96	2.04		4	8.2	10.6	2.37	3.76	
Age	≤31	0	2	5	0.64	1.64	0.664	0	2	4	0.64	1.56	0.295	1	5	7	1.27	2.77	0.404
	>31	0	2	5	0.59	1.64		0	2	4	0.54	1.49		1	4	8	1.13	2.76	
Mental health (14 months after birth)	≤9	0	1	3	0.39	1.15	**0.015**	0	2	3	0.46	1.2	0.265	1	3	5	0.85	2	**0.040**
	>9	0	2	5	0.68	1.8		0	2	3	0.58	1.55		1	4	8	1.26	2.98	
Parity	0	0	2	5	0.61	1.67	0.921	0	2	4	0.6	1.57	0.833	1	5	8	1.22	2.84	0.954
	≥1	0	2	4.05	0.62	1.6		0	2	4	0.59	1.48		1	4	7	1.21	2.65	
Smoking in pregnancy	No	0	2	4	0.54	1.54	**0.003**	0	2	3.45	0.53	1.45	**0.006**	1	4	6	1.08	2.6	**0.001**
	Yes	1	4.4	6	1.06	2.11		1	4	5	0.97	1.93		2	7	10	2.03	3.51	
Country of birth	Spain	0	2	5	0.6	1.61	0.305	0	2	4	0.59	1.51	0.430	1	4	7.8	1.19	2.72	0.292
	Other	1	2.3	6.3	0.87	2.08		1	2	5	0.76	1.81		2	5	7.3	1.63	3.38	
Social class	Non-manual	0	1	3	0.44	1.31	**<0.001**	0	2	3	0.54	1.38	0.158	1	3	6	0.98	2.36	**0.002**
	Manual	0	3	6	0.83	1.94		0	2	4	0.67	1.69		1	5	9	1.5	3.15	
Education level	Primary	0.25	5	7	1.03	2.25	**<0.001**	0.25	2	4	0.7	1.68	0.283	2	6.3	10	1.73	3.41	**0.001**
	Secondary	0	2	4	0.61	1.52		0	2	4	0.62	1.54		1	5	6	1.23	2.64	
	University	0	1	3	0.39	1.28		0	2	3	0.51	1.41		0	3	5	0.9	2.41	

* t-test for mean comparison (2 samples) or ANOVA (more than two samples).

**Table 3:** Zero-inflated Poisson regression models for the association between inattention, hyperactivity and ADHD symptoms with ferritin levels (mg/L log scale) in continuous scale and in tertiles.

		Tertile 1 [1.98, 20.92] (mg/L)	Tertile 2 (20.92, 38.79] (mg/L)	Tertile 3 (38.79, 216.5] (mg/L)	p for Trend **	Continuous
		Beta CI (95%)	Beta CI (95%)	Beta CI (95%)		Beta CI (95%)
*Inattention*						
	Count model *^1^					
	Male ***	1 (Ref.)	**−0.3 (−0.55, −0.05)**	**−0.37 (−0.6, −0.14)**	*0.002*	**−0.19 (−0.32, −0.07)**
	Female ***	1 (Ref.)	−0.11 (−0.64, 0.42)	0.35 (−0.07, 0.77)	*0.066*	0.09 (−0.13, 0.31)
	Zero-inflation model *^2^	1 (Ref.)	0.19 (−0.24, 0.63)	0.19 (−0.24, 0.63)	*0.488*	−0.04 (−0.26, 0.19)
*Hyperactivity*						
	Count model *^1^	1 (Ref.)	−0.07 (−0.29, 0.16)	−0.11 (−0.33, 0.11)	*0.327*	−0.03 (−0.15, 0.09)
	Zero-inflation model *^2^	1 (Ref.)	0.24 (−0.16, 0.64)	0.17 (−0.23, 0.56)	*0.411*	0.11 (−0.11, 0.32)
*ADHD*						
	Count model *^1^	1 (Ref.)	−0.09 (−0.24, 0.06)	−0.09 (−0.23, 0.05)	*0.239*	−0.02 (−0.1, 0.05)
	Zero-inflation model *^2^	1 (Ref.)	0.36 (0, 0.71)	0.12 (−0.22, 0.47)	*0.515*	0.13 (−0.06, 0.33)
						

Models adjusted by cohort, smoking during pregnancy, alcohol intake during pregnancy, pre-pregnancy BMI, social class, sex and age of the child, living with mother/father and weeks (ferritin extraction). *^1^ Count model coefficients (Poisson with log link). *^2^ Zero−inflation model coefficients (binomial with logit link). ** *p* for the trend between tertiles. *** Model with interaction term between sex and ferritin.

**Table 4:** Zero-inflated Poisson regression models for the association between inattention, hyperactivity and ADHD symptoms with ferritin levels (log scale).

		Tertile 1	Tertile 2	Tertile 3	p Trend **	Continuous
		[1.98, 20.92] (mg/L)	(20.92, 38.79] (mg/L)	(38.79, 216.5] (mg/L)		
		Beta CI (95%)	Beta CI (95%)	Beta CI (95%)		Beta CI (95%)
**Stratified by sex**						
*Inattention*						
Boys	Count model *^1^	1 (Ref.)	**−0.32 (−0.57, −0.06)**	**−0.4 (−0.63, −0.16)**	***0.001***	**−0.21 (−0.34, −0.08)**
	Zero-inflation model *^2^	1 (Ref.)	0.09 (−0.46, 0.63)	−0.27 (−0.8, 0.25)	*0.27*	−0.14 (−0.43, 0.15)
Girls	Count model*^1^	1 (Ref.)	0.04 (−0.61, 0.69)	**0.58 (0.02, 1.13)**	***0.012***	0.15 (−0.09, 0.39)
	Zero-inflation model *^2^	1 (Ref.)	0.37 (−0.36, 1.09)	−0.32 (−1.13, 0.49)	*0.311*	−0.38 (−1.17, 0.42)
*Hyperactivity*						
Boys	Count model *^1^	1 (Ref.)	−0.09 (−0.37, 0.19)	−0.16 (−0.44, 0.11)	*0.244*	−0.03 (−0.18, 0.12)
	Zero-inflation model *^2^	1 (Ref.)	0.16 (−0.37, 0.68)	0 (−0.52, 0.51)	*0.981*	−0.03 (−0.32, 0.25)
Girls	Count model *^1^	1 (Ref.)	−0.02 (−0.44, 0.39)	−0.07 (−0.49, 0.35)	*0.727*	−0.1 (−0.33, 0.13)
	Zero-inflation model *^2^	1 (Ref.)	0.41 (−0.22, 1.04)	0.48 (−0.16, 1.12)	*0.143*	0.34 (−0.02, 0.7)
*ADHD*						
Boys	Count model *^1^	1 (Ref.)	−0.14 (−0.32, 0.03)	**−0.22 (−0.39, −0.06)**	***0.008***	−0.07 (−0.16, 0.02)
	Zero-inflation model *^2^	1 (Ref.)	0.25 (−0.23, 0.73)	−0.05 (−0.52, 0.41)	*0.778*	−0.02 (−0.27, 0.24)
Girls	Count model *^1^	1 (Ref.)	0.02 (−0.31, 0.34)	0.17 (−0.13, 0.47)	*0.237*	0.02 (−0.14, 0.19)
	Zero-inflation model *^2^	1 (Ref.)	**0.6 (0.05, 1.16)**	0.49 (−0.05, 1.04)	*0.077*	**0.39 (0.08, 0.69)**
**Stratified by cohort**						
*Inattention*						
Gipuzkoa	Count model *^1^	1 (Ref.)	−0.18 (−0.82, 0.47)	0.18 (−0.35, 0.71)	*0.41*	0.22 (−0.04, 0.48)
	Zero-inflation model *^2^	1 (Ref.)	0.8 (−0.15, 1.75)	0.05 (−0.82, 0.91)	*0.873*	0.16 (−0.31, 0.63)
Sabadell	Count model *^1^	1 (Ref.)	−0.35 (−0.72, 0.03)	−0.22 (−0.55, 0.1)	*0.116*	**−0.16 (−0.31, 0)**
	Zero-inflation model *^2^	1 (Ref.)	0.38 (−0.29, 1.05)	0.03 (−0.6, 0.66)	*0.887*	−0.03 (−0.36, 0.3)
Valencia	Count model *^1^	1 (Ref.)	−0.24 (−0.64, 0.15)	**−0.39 (−0.76, −0.02)**	***0.043***	**−0.29 (−0.51, −0.06)**
	Zero-inflation model *^2^	1 (Ref.)	−0.44 (−1.22, 0.35)	−0.6 (−1.35, 0.14)	*0.123*	−0.27 (−0.71, 0.18)
*Hyperactivity*						
Gipuzkoa	Count model *^1^	1 (Ref.)	−0.07 (−0.77, 0.62)	0.06 (−0.67, 0.78)	*0.932*	0.23 (−0.11, 0.57)
	Zero-inflation model *^2^	1 (Ref.)	0.65 (−0.3, 1.6)	−0.1 (−1, 0.8)	*0.838*	0.1 (−0.4, 0.59)
Sabadell	Count model *^1^	1 (Ref.)	−0.25 (−0.6, 0.11)	−0.31 (−0.64, 0.02)	*0.053*	−0.12 (−0.28, 0.03)
	Zero-inflation model *^2^	1 (Ref.)	0.24 (−0.36, 0.85)	−0.09 (−0.69, 0.51)	*0.814*	−0.04 (−0.35, 0.27)
Valencia	Count model *^1^	1 (Ref.)	0.06 (−0.36, 0.48)	0.06 (−0.36, 0.48)	*0.904*	0.01 (−0.25, 0.27)
	Zero-inflation model *^2^	1 (Ref.)	−0.02 (−0.87, 0.82)	0.59 (−0.17, 1.36)	*0.07*	0.4 (−0.03, 0.82)
*ADHD*						
Gipuzkoa	Count model *^1^	1 (Ref.)	−0.06 (−0.48, 0.36)	0.37 (−0.01, 0.74)	***0.045***	**0.33 (0.14, 0.53)**
	Zero-inflation model *^2^	1 (Ref.)	**0.85 (0.06, 1.63)**	0.27 (−0.48, 1.02)	*0.412*	0.28 (−0.13, 0.69)
Sabadell	Count model *^1^	1 (Ref.)	**−0.29 (−0.53, −0.06)**	**−0.28 (−0.5, −0.07)**	***0.005***	−0.1 (−0.21, 0)
	Zero-inflation model *^2^	1 (Ref.)	0.39 (−0.14, 0.92)	0.03 (−0.49, 0.55)	*0.857*	0.08 (−0.19, 0.35)
Valencia	Count model *^1^	1 (Ref.)	0.09 (−0.16, 0.35)	−0.11 (−0.35, 0.14)	*0.244*	−0.07 (−0.21, 0.08)
	Zero-inflation model *^2^	1 (Ref.)	−0.04 (−0.68, 0.6)	0.07 (−0.55, 0.69)	*0.788*	0.12 (−0.24, 0.49)

Models adjusted by cohort, smoking during pregnancy, alcohol intake during pregnancy, pre-pregnancy BMI, social class, sex and age of the child, living with mother/father and weeks (ferritin extraction). Gipuzkoa and Sabadell models do not include the variable “lives with mother/father”. *^1^ Count model coefficients (Poisson with log link). *^2^ Zero-inflation model coefficients (binomial with logit link). ** *p* for the trend between tertiles.

## Supplementary Materials

The following are available online at https://www.mdpi.com/1660-4601/17/21/7704/s1, Table S1: Descriptive characteristics of the study sample and the excluded sample.
