# Correction: Santa-Marina et al. Maternal Ferritin Levels during Pregnancy and ADHD Symptoms in 4-Year-Old Children: Results from the INMA-INfancia y Medio Ambiente (Environment and Childhood) Prospective Birth Cohort Study. *Int. J. Environ. Res. Public Health* 2020, *17*, 7704

**DOI:** 10.3390/ijerph18126283

**Published:** 2021-06-10

**Authors:** Loreto Santa-Marina, Nerea Lertxundi, Ainara Andiarena, Amaia Irizar, Jordi Sunyer, Amaia Molinuevo, Sabrina Llop, Jordi Julvez, Andrea Beneito, Jesús Ibarluzea, Liher Imaz, Maite Ferrin

**Affiliations:** 1 Spanish Consortium for Research on Epidemiology and Public Health (CIBERESP), Instituto de Salud Carlos III, C/Monforte de Lemos 3–5, 28029 Madrid, Spain; ambien4ss-san@euskadi.eus (L.S.-M.); jordi.sunyer@isglobal.org (J.S.); au-molinuevo@euskadi.eus (A.M.); llop_sab@gva.es (S.L.); jordi.julvez@isglobal.org (J.J.); mambien3-san@euskadi.eus (J.I.); 2 Biodonostia, Epidemiology and Public Health Area, Environmental Epidemiology and Child Development Group, 20014 San Sebastian, Spain; nerea.lertxundi@ehu.eus (N.L.); ainara.andiarena@ehu.eus (A.A.); 3 Public Health Division of Gipuzkoa, Basque Government, 20013 San Sebastian, Spain; l-imazgoienetxea@euskadi.eus; 4 Faculty of Psychology, University of the Basque Country (UPV/EHU), Avenida Tolosa 70, 20018 San Sebastian, Spain; 5 Faculty of Medicine and Nursing, University of the Basque Country (UPV/EHU), Barrio Sarriena s/n, 48940 Leioa, Spain; 6 Hospital del Mar Research Institute, 08003 Barcelona, Spain; 7 ISGlobal—Instituto de Salud Global de Barcelona–Campus MAR, PRBB, 08003 Barcelona, Spain; 8 Epidemiology and Environmental Health Joint Research Unit, FISABIO–Universitat Jaume I–Universitat de València, 08003 València, Spain; beneito_and@gva.es; 9 Institut d’Investigació Sanitària Pere Virgili (IISPV), Hospital Universitari Sant Joan de Reus, 43204 Reus, Spain; 10 Biodonostia, Epidemiology and Public Health Area, Epidemiology of Chronic and Communicable Diseases Group, 20014 San Sebastian, Spain; 11 Haringey Child and Adolescent Mental Health Service, Barnet, Enfield and Haringey NHS Mental Health Trust, London N15 3TH, UK; maiteferrin@yahoo.es; 12 Recognition Health, London W1G 9RU, UK

## 1. Error in Table 3

In the original article [1], there was a mistake in Table 3 as published. The units displayed for ferritin (mg/L) are incorrect, with µg/L being the correct unit for that parameter. Values for ferritin levels are correct, and only the units are affected by this typographic mistake. Interpretation of the results are not affected, since it was carried out with the correct values and units from the analysis. The corrected version of Table 3 appears below:

**Table 3:** Zero-inflated Poisson regression models for the association between inattention, hyperactivity, and ADHD symptoms with ferritin levels (µg/L log scale) on a continuous scale and in tertiles.

		Tertile 1 [1.98, 20.92] (µg/L)	Tertile 2 (20.92, 38.79] (µg/L)	Tertile 3 (38.79, 216.5] (µg/L)	p for Trend **	Continuous
		Beta CI (95%)	Beta CI (95%)	Beta CI (95%)		Beta CI (95%)
*Inattention*						
	Count model *^1^					
	Male ***	1 (Ref.)	**−0.3 (−0.55, −0.05)**	**−0.37 (−0.6, −0.14)**	**0.002**	**−0.19 (−0.32, −0.07)**
	Female ***	1 (Ref.)	−0.11 (−0.64, 0.42)	0.35 (−0.07, 0.77)	0.066	0.09 (−0.13, 0.31)
	Zero-inflation model *^2^	1 (Ref.)	0.19 (−0.24, 0.63)	0.19 (−0.24, 0.63)	0.488	−0.04 (−0.26, 0.19)
*Hyperactivity*						
	Count model *^1^	1 (Ref.)	−0.07 (−0.29, 0.16)	−0.11 (−0.33, 0.11)	0.327	−0.03 (−0.15, 0.09)
	Zero-inflation model *^2^	1 (Ref.)	0.24 (−0.16, 0.64)	0.17 (−0.23, 0.56)	0.411	0.11 (−0.11, 0.32)
*ADHD*						
	Count model *^1^	1 (Ref.)	−0.09 (−0.24, 0.06)	−0.09 (−0.23, 0.05)	0.239	−0.02 (−0.1, 0.05)
	Zero-inflation model *^2^	1 (Ref.)	0.36 (0, 0.71)	0.12 (−0.22, 0.47)	0.515	0.13 (−0.06, 0.33)

Models adjusted by cohort, smoking during pregnancy, alcohol intake during pregnancy, pre-pregnancy BMI, social class, sex and age of the child, living with mother/father, and weeks (week of sample collection). *^1^ Count model coefficients (Poisson with log link). *^2^ Zero-inflation model coefficients (binomial with logit link). ** *p* for the trend between tertiles. *** Model with interaction term between sex and ferritin. Significant associations (*p* < 0.05) are indicated in bold.

## 2. Error in Table 4

In the original article [1], there was a mistake in Table 4 as published. The units displayed for ferritin (mg/L) are incorrect, with µg/L being the correct unit for that parameter. Values for ferritin levels are correct, and only the units are affected by this typographic mistake. Interpretation of the results are not affected, since it was carried out with the correct values and units from the analysis. The corrected version of Table 4 appears below:

**Table 4:** Zero-inflated Poisson regression models for the association between inattention, hyperactivity, and ADHD symptoms with ferritin levels (log scale).

		Tertile 1 [1.98, 20.92] (µg/L)	Tertile 2 (20.92, 38.79] (µg/L)	Tertile 3 (38.79, 216.5] (µg/L)	p for Trend **	Continuous
		Beta CI (95%)	Beta CI (95%)	Beta CI (95%)		Beta CI (95%)
**stratified by Sex**						
*Inattention*						
Boys	Count model *^1^	1 (Ref.)	**−0.32 (−0.57, −0.06)**	**−0.4 (−0.63, −0.16)**	**0.001**	**−0.21 (−0.34, −0.08)**
	Zero-inflation model *^2^	1 (Ref.)	0.09 (−0.46, 0.63)	−0.27 (−0.8, 0.25)	0.270	−0.14 (−0.43, 0.15)
Girls	Count model *^1^	1 (Ref.)	0.04 (−0.61, 0.69)	**0.58 (0.02, 1.13)**	**0.012**	0.15 (−0.09, 0.39)
	Zero-inflation model *^2^	1 (Ref.)	0.37 (−0.36, 1.09)	−0.32 (−1.13, 0.49)	0.311	−0.38 (−1.17, 0.42)
*Hyperactivity*						
Boys	Count model *^1^	1 (Ref.)	−0.09 (−0.37, 0.19)	−0.16 (−0.44, 0.11)	0.244	−0.03 (−0.18, 0.12)
	Zero-inflation model *^2^	1 (Ref.)	0.16 (−0.37, 0.68)	0 (−0.52, 0.51)	0.981	−0.03 (−0.32, 0.25)
Girls	Count model *^1^	1 (Ref.)	−0.02 (−0.44, 0.39)	−0.07 (−0.49, 0.35)	0.727	−0.1 (−0.33, 0.13)
	Zero-inflation model *^2^	1 (Ref.)	0.41 (−0.22, 1.04)	0.48 (−0.16, 1.12)	0.143	0.34 (−0.02, 0.7)
*ADHD*						
Boys	Count model *^1^	1 (Ref.)	−0.14 (−0.32, 0.03)	**−0.22 (−0.39, −0.06)**	**0.008**	−0.07 (−0.16, 0.02)
	Zero-inflation model *^2^	1 (Ref.)	0.25 (−0.23, 0.73)	−0.05 (−0.52, 0.41)	0.778	−0.02 (−0.27, 0.24)
Girls	Count model *^1^	1 (Ref.)	0.02 (−0.31, 0.34)	0.17 (−0.13, 0.47)	0.237	0.02 (−0.14, 0.19)
	Zero-inflation model *^2^	1 (Ref.)	**0.6 (0.05, 1.16)**	0.49 (−0.05, 1.04)	0.077	**0.39 (0.08, 0.69)**
**Stratified by Cohort**						
*Inattention*						
Gipuzkoa	Count model *^1^	1 (Ref.)	−0.18 (−0.82, 0.47)	0.18 (−0.35, 0.71)	0.410	0.22 (−0.04, 0.48)
	Zero-inflation model *^2^	1 (Ref.)	0.8 (−0.15, 1.75)	0.05 (−0.82, 0.91)	0.873	0.16 (−0.31, 0.63)
Sabadell	Count model *^1^	1 (Ref.)	−0.35 (−0.72, 0.03)	−0.22 (−0.55, 0.1)	0.116	**−0.16 (−0.31, 0)**
	Zero-inflation model *^2^	1 (Ref.)	0.38 (−0.29, 1.05)	0.03 (−0.6, 0.66)	0.887	−0.03 (−0.36, 0.3)
Valencia	Count model *^1^	1 (Ref.)	−0.24 (−0.64, 0.15)	**−0.39 (−0.76, −0.02)**	**0.043**	**−0.29 (−0.51, −0.06)**
	Zero-inflation model *^2^	1 (Ref.)	−0.44 (−1.22, 0.35)	−0.6 (−1.35, 0.14)	0.123	−0.27 (−0.71, 0.18)
*Hyperactivity*						
Gipuzkoa	Count model *^1^	1 (Ref.)	−0.07 (−0.77, 0.62)	0.06 (−0.67, 0.78)	0.932	0.23 (−0.11, 0.57)
	Zero-inflation model *^2^	1 (Ref.)	0.65 (−0.3, 1.6)	−0.1 (−1, 0.8)	0.838	0.1 (−0.4, 0.59)
Sabadell	Count model *^1^	1 (Ref.)	−0.25 (−0.6, 0.11)	−0.31 (−0.64, 0.02)	0.053	−0.12 (−0.28, 0.03)
	Zero-inflation model *^2^	1 (Ref.)	0.24 (−0.36, 0.85)	−0.09 (−0.69, 0.51)	0.814	−0.04 (−0.35, 0.27)
Valencia	Count model *^1^	1 (Ref.)	0.06 (−0.36, 0.48)	0.06 (−0.36, 0.48)	0.904	0.01 (−0.25, 0.27)
	Zero-inflation model *^2^	1 (Ref.)	−0.02 (−0.87, 0.82)	0.59 (−0.17, 1.36)	0.070	0.4 (−0.03, 0.82)
*ADHD*						
Gipuzkoa	Count model *^1^	1 (Ref.)	−0.06 (−0.48, 0.36)	0.37 (−0.01, 0.74)	**0.045**	**0.33 (0.14, 0.53)**
	Zero-inflation model *^2^	1 (Ref.)	**0.85 (0.06, 1.63)**	0.27 (−0.48, 1.02)	0.412	0.28 (−0.13, 0.69)
Sabadell	Count model *^1^	1 (Ref.)	**−0.29 (−0.53, −0.06)**	**−0.28 (−0.5, −0.07)**	**0.005**	−0.1 (−0.21, 0)
	Zero-inflation model *^2^	1 (Ref.)	0.39 (−0.14, 0.92)	0.03 (−0.49, 0.55)	0.857	0.08 (−0.19, 0.35)
Valencia	Count model *^1^	1 (Ref.)	0.09 (−0.16, 0.35)	−0.11 (−0.35, 0.14)	0.244	−0.07 (−0.21, 0.08)
	Zero-inflation model *^2^	1 (Ref.)	−0.04 (−0.68, 0.6)	0.07 (−0.55, 0.69)	0.788	0.12 (−0.24, 0.49)

Models adjusted by cohort, smoking during pregnancy, alcohol intake during pregnancy, pre-pregnancy BMI, social class, sex and age of the child, living with mother/father, and weeks (week of sample collection). For Gipuzkoa and Sabadell, models do not include the variable “lives with mother/father”. *^1^ Count model coefficients (Poisson with log link). *^2^ Zero-inflation model coefficients (binomial with logit link). ** *p* for the trend between tertiles. Significant associations (*p* < 0.05) are indicated in bold.

## 3. Text Correction

In the original article, the units mentioned in the abstract regarding ferritin levels were also incorrect. Hence, we corrected Abstract lines 8–10: “Comparing ferritin level by tertile, significant differences were observed between the first tertile (1.98, 20.92) and the second (20.92, 38.79) and third (38.79, 216.5) (µg/L) tertiles”.

The authors apologize for any inconvenience caused and state that the scientific conclusions are unaffected. The original article has been updated.
