# Supplementary material for: Maternal Ferritin Levels during Pregnancy and ADHD Symptoms in 4-Year-Old Children: Results from the INMA–INfancia y Medio Ambiente (Environment and Childhood) Prospective Birth Cohort Study
Source: Int J Environ Res Public Health. 2020 Oct 22;17(21):7704. doi: 10.3390/ijerph17217704 (PMC7659477; doi:10.3390/ijerph17217704)
Supplement: Supplementary file 1 [file ijerph-17-07704-s001.pdf]

Table S1. Descriptive characteristics of the study simple and the excluded sample.

		TOTAL N = 1095		EXCLUDED N = 1055		*p*
Variables		N (%)	Mean(SD)	N (%)	Mean(SD)	
Child characteristics						
Sex	Female	540 (49.32)		441 (41.8)		*0.551*
	Male	555 (50.68)		480 (45.5)		
	Missing	*0 (0)*		*134 (12.7)*		
Preterm	No	1046 (95.53)		869 (82.4)		*0,171*
	Yes	41 (3.74)		47 (4.45)		
	Missing	*8 (0.73)*		*139 (13.2)*		
Sibling order	Not first	478 (43.65)		553 (52.42)		*0,304*
	First	617 (56.35)		472 (44.74)		
	Missing	*0 (0)*		*30 (2.84)*		
Lives with mother/father	Both	1053 (96.16)		715 (67.77)		
	Only one	15 (1.37)		15 (1.42)		
	Missing	*27 (2.47)*		325 (30.81)		*0,3844*
Number of people who lives with	*Missing*	*27 (2.47)*	2.57 (0.76)	*325 (30.81)*	2.70 (0.98)	***0,004***
Breastfeeding	*Missing*	*14 (1.28)*	26.6 (19.82)	*283 (26.82)*	24.9 (20.1)	*0,065*
Hg (μg/l) in umbilical cord	*Missing*	*247 (22.5)*	10.77 (8.65)	*357 (33.84)*	9.79 (8.52)	***0,026***
Maternal characteristics						
Ferritin level (log scale)	*Missing*	*0 (0)*	3.32 (0.75)	*196 (18.58)*	3.29 (0.75)	
Maternal blood collection week	*Missing*	*2 (0.18)*	11.57 (3.58)	*68 (6.45)*	12.69 (7.01)	***<0.001***
Alcohol intake during pregnancy	*Missing*	*0 (0)*	0.33 (0.98)	*20 (2.75)*	0.37 (1.19)	*0,46*
Pre-pregnancy BMI	*Missing*	*0 (0)*	23.57 (4.29)	*31 (2.94)*	23.4 (4.36)	*0,5*
Age	*Missing*	*1 (0.09)*	30.92 (3.94)	*0 (0)*	29.8 (4.74)	***<0.001***
Mental health (14 months after birth)	*Missing*	*405 (36.99)*	9.64 (3.72)	*730 (69.19)*	9.95 (4.39)	*0,249*
Parity	*Missing*	*2 (0.18)*	0.51 (0.64)	*30 (2.84)*	0.56 (0.71)	*0,061*
Smoking during pregnancy	No	912 (83.29)		730 (69.2)		*0,082*
	Yes	167 (15.25)		166 (15.7)		
	Missing	16 (1.46)		*159 (15.1)*		
Country of birth	Spain	1025 (93.61)		795 (75.36)		***<0.001***
	Other	68 (6.21)		117 (11.1)		
	Missing	2 (0.18)		*143 (13.6)*		
Social class	Non manual	594 (54.25)		379 (35.9)		***<0.001***
	Manual	501 (45.75)		539 (51.1)		
	Missing	*0 (0)*		*137 (13.0)*		
Education level	Primary	228 (20.82)		335 (31.8)		***<0.001***
	Secondary	461 (42.1)		401 (38.0)		
	University	402 (36.71)		289 (27.4)		
	Missing	4 (0.37)		*30 (2.84)*		

* One-way ANOVA for mean diferences and Chi squared test for categorical variables.
